# Cross-sectional study on ethnic variations in gut microbiome diversity and hypertension severity

**DOI:** 10.6026/973206300213043

**Published:** 2025-09-30

**Authors:** Rajput Nixitsinh Arvindssinh, Tanvi Ghanshyambhai Patel, M.R Madan Karthik Raj

**Affiliations:** 1 Department of Physiology, Junior Resident at GMERS Gotri Medical College and Hospital, Vadodara, Gujarat, India; 2 Department of Anatomy, Junior Resident at GMERS, Gotri Medical College and Hospital, Vadodara, Gujarat, India; 3 Department of General Surgery, Vinayaka MIssion's Kirupanandha Variyar Medical College and Hospital, Salem, Tamilnadu, India

**Keywords:** Gut microbiome, hypertension, ethnic variations, microbial diversity, 16S rRNA sequencing

## Abstract

Stool samples were analyzed using 16S rRNA sequencing and blood pressure measurements were categorized per AHA guidelines. Hence, 130 adults from three ethnic groups to assess differences in gut microbiome diversity and their association with hypertension severity were studied. Significant ethnic variations were observed in microbial composition, particularly in Firmicutes-to-Bacteroidetes ratios. Greater microbial diversity was associated with lower hypertension severity in certain ethnicities. Thus, we show ethnic-specific microbiome patterns may influence hypertension outcomes.

## Background:

Hypertension remains a major global health burden, contributing significantly to cardiovascular morbidity and mortality [1]. Despite widespread awareness and treatment options, blood pressure control rates vary markedly across different ethnic populations, hinting at complex underlying mechanisms beyond conventional risk factors [2]. Recent research highlights the gut microbiome as a potential modulator of blood pressure, given its role in metabolic regulation, immune function and production of bioactive metabolites such as short-chain fatty acids [3]. Emerging studies suggest that the composition and diversity of gut microbiota vary significantly across ethnic groups due to genetic, dietary, environmental and cultural differences [4]. These microbial differences may influence susceptibility to hypertension or affect its severity through mechanisms like inflammation, endothelial dysfunction and sodium absorption [5]. However, limited research has comprehensively explored how ethnic variation in the gut microbiome correlates with hypertension severity in a diverse population [6]. Therefore, it is of interest to bridge this gap by examining the association between gut microbiome diversity and hypertension severity across multiple ethnic groups in a well-defined adult population. Understanding these interactions could pave the way for ethnicity-tailored interventions and microbiome-based therapeutic strategies for blood pressure management.

## Materials and Methods:

This cross-sectional study recruited 130 adult participants aged 30-65 years from three self-identified ethnic groups: Group A (n=45), Group B (n=42) and Group C (n=43). Participants were selected from outpatient clinics and community health centers, with inclusion criteria being a confirmed diagnosis of hypertension and no recent antibiotic or probiotic use within the past three months. After obtaining informed consent, demographic and clinical data were collected through structured interviews and electronic medical records. Blood pressure was measured using a calibrated sphygmomanometer and hypertension severity was categorized based on the American Heart Association (AHA) guidelines into Stage 1, Stage 2 and Hypertensive Crisis. Fecal samples were self-collected by participants using sterile containers and stored at -80°C until analysis. Microbial DNA was extracted using a standard QIAamp DNA Stool Mini Kit protocol, followed by 16S rRNA gene sequencing targeting the V3-V4 regions on the Illumina MiSeq platform. Sequences were processed using QIIME2 for operational taxonomic unit (OTU) picking and microbial diversity analysis. Alpha and beta diversity metrics, including Shannon index and UniFrac distances, were calculated. Statistical comparisons between ethnic groups and hypertension categories were performed using ANOVA, Kruskal-Wallis and multivariate regression models, adjusting for age, sex, BMI and dietary intake.

## Results:

This study analyzed gut microbiome diversity and its association with hypertension severity across 130 participants divided into three ethnic groups. Significant inter-ethnic variations were observed in microbial composition, alpha diversity indices and specific bacterial taxa. A negative correlation was noted between microbial diversity and hypertension severity, particularly within Group A. Table 1 (see PDF) shows demographic characteristics were generally balanced across the ethnic groups, with slight variations in mean BMI and age. Group C showed a marginally higher prevalence of Stage 2 hypertension. Table 2 (see PDF) shows alpha diversity, measured by Shannon index, was significantly higher in Group A compared to Group C, suggesting greater microbial richness and evenness in Group A. Table 3 (see PDF) shows beta diversity based on weighted UniFrac distances showed clear clustering by ethnicity, indicating distinct microbial community compositions across groups. Table 4 (see PDF) shows the Firmicutes-to-Bacteroidetes ratio was highest in Group C, potentially contributing to higher hypertension severity in this group. Table 5 (see PDF) shows higher levels of short-chain fatty acid (SCFA)-producing bacteria were observed in participants with Stage 1 hypertension compared to those with more severe hypertension. Table 6 (see PDF) shows participants with higher microbial diversity had significantly lower mean systolic and diastolic blood pressure. Table 7 (see PDF) shows BMI was positively correlated with hypertension severity but inversely related to microbial diversity, suggesting obesity may mediate microbiome-blood pressure interactions. Table 8 shows specific genera such as Prevotella and Clostridium varied significantly between ethnic groups, indicating potential biomarkers for ethnicity-based stratification. Table 9 (see PDF) shows Dietary fiber intake was positively correlated with both microbial diversity and presence of beneficial SCFA-producing bacteria. Table 10 (see PDF) shows regression analysis showed microbial diversity independently predicted lower hypertension severity after adjusting for confounders.

## Discussion:

This study explored the ethnic variability in gut microbiome diversity and its relationship with hypertension severity in a sample of 130 hypertensive adults. The findings revealed significant inter-ethnic differences in both microbial composition and diversity metrics. Notably, Group A demonstrated higher alpha diversity, which was inversely associated with blood pressure levels and hypertension severit. These results reinforce the emerging view that a more diverse gut microbiome may play a protective role against the progression of hypertension, likely through mechanisms involving metabolic regulation, reduced systemic inflammation and enhanced vascular function [7]. The elevated Firmicutes-to-Bacteroidetes ratio in Group C, along with higher prevalence of Stage 2 hypertension in this group, aligns with prior research suggesting that dysbiosis characterized by a shift toward Firmicutes dominance can promote pro-inflammatory states and impair gut barrier integrity [8]. Additionally, reduced abundance of SCFA-producing bacteria such as Faecalibacterium prausnitzii and Roseburia in participants with more severe hypertension suggests a possible mechanistic link between microbial metabolite profiles and vascular health. These beneficial bacteria are known to produce butyrate, which improves endothelial function, modulates immune response and reduces oxidative stress-all relevant to hypertension pathophysiology [9, 10]. Importantly, the observed relationship between dietary fiber intake and microbial diversity underscores the modifiable nature of the gut microbiome. Participants with higher fiber intake showed greater abundance of beneficial microbes and lower blood pressure readings, suggesting that dietary interventions could serve as an accessible, non-pharmacological strategy for managing hypertension, especially when tailored to ethnic-specific microbiome patterns [11, 12]. While the cross-sectional design limits causal inference, the multivariate regression analysis adjusted for major confounders such as BMI, age and diet, thereby strengthening the validity of associations found [13, 14]. Future longitudinal and interventional studies are warranted to clarify causal relationships and to test whether microbiome-targeted therapies yield differential outcomes based on ethnic background. Overall, this study highlights the need to consider ethnic microbiome profiles in personalized hypertension management and adds to the growing body of evidence linking gut health with cardiovascular disease.

## Conclusion:

Ethnic differences in gut microbiome diversity and their association with hypertension severity is shown. Greater microbial diversity and higher abundance of SCFA-producing bacteria were linked to lower blood pressure. Thus, we show the potential for ethnicity-informed, microbiome-targeted interventions in hypertension management.
